# Supplementary material for: Molecular, anatomical, and functional organization of lung interoceptors
Source: bioRxiv. 2025 Nov 13:2021.11.10.468116. Preprint. [Version 2] doi: 10.1101/2021.11.10.468116 (PMC12642431; doi:10.1101/2021.11.10.468116)
Supplement: 8 [file NIHPP2021.11.10.468116v2-supplement-8.pdf]

bioRxiv preprint doi: https://doi.org/10.1101/2021.11.10.468116; this version posted November 13, 2025. The copyright holder for this preprint (which was not certified by peer review) is the author/funder, who has granted bioRxiv a license to display the preprint in perpetuity. It is made available under aCC-BY 4.0 International license.

Figure S1

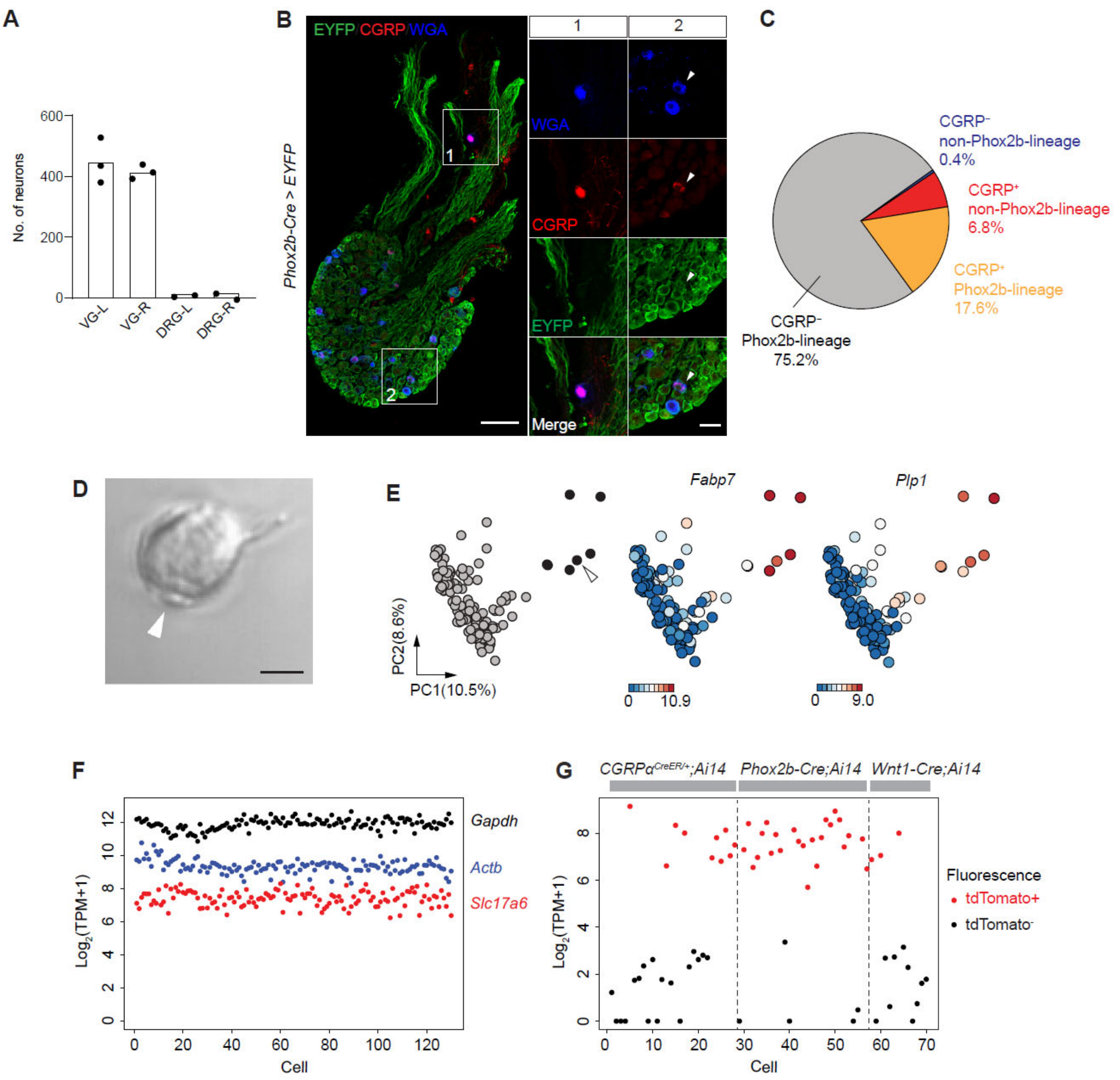

bioRxiv preprint doi: https://doi.org/10.1101/2021.11.10.468116; this version posted November 13, 2025. The copyright holder for this preprint (which was not certified by peer review) is the author/funder, who has granted bioRxiv a license to display the preprint in perpetuity. It is made available under aCC-BY 4.0 International license.

Figure S1. Single-cell RNA-seq of vagal PSNs, related to Figure 1

(A) Quantification of WGA-labeled neurons in vagal (VG) and dorsal root ganglia (DRG, C7-T6 collectively) on the side of the body indicated (L, left, R, right). Dots, values from individual mice; bars, mean values.

(B) A vagal ganglion section from a *Phox2b-Cre;*Ai32/+ mouse with WGA labeling from the lungs, immunostained for indicated markers. Close-ups of boxed areas are shown in split panels on the right. Box 1, a CGRP^{+} Phox2b-lineage negative (EYFP^{-}) WGA-labeled neuron. Box 2, a CGRP^{+} Phox2b-lineage positive (EYFP^{+}) WGA-labeled neuron (arrowhead). Scale bars: 100 µm, 20 µm (insets).

(C) Fractions of WGA-labeled vagal sensory neurons with indicated combinations of CGRP and Phox2b-lineage positivity (n=1122 scored neurons in 3 ganglia from 3 mice).

(D) Brightfield image of a PSN with satellite glial cell(s) still attached (arrowhead) after ganglion cell dissociation. Scale bar: 10 µm.

(E) PC plots showing 7 cells (black) separated from others (gray) when satellite glial cell-enriched genes were used for analysis. These cells expressed high levels of glial cell marker genes *Fabp7* and *Plp1*. Expression scale: $\log_2$(TPM+1) for range indicated. Arrowhead, cell shown in (D).

(G) Sequencing results of housekeeping genes (*Gapdh*, *Actb*) and a sensory neuron marker gene (*Slc17a6*) across all cells passed quality control. High consistency and no drop-out indicate high quality expression profiles.

(H) Sequencing results of *tdTomato* expression in cells picked from mice with genetic labeling as indicated. Red and black dots, cells with (red) and without (black) tdTomato fluorescence observed during cell picking. Note sequencing results were consistent with the fluorescence records. Low levels of tdTomato reads detected in some of the fluorescence-negative cells are likely due to low-level leaky

bioRxiv preprint doi: https://doi.org/10.1101/2021.11.10.468116; this version posted November 13, 2025. The copyright holder for this preprint (which was not certified by peer review) is the author/funder, who has granted bioRxiv a license to display the preprint in perpetuity. It is made available under aCC-BY 4.0 International license.

expression of the reporter gene or contaminating mRNA released from dying or dead tdTomato-expressing cells, given that it was not observed in neurons from wild type mice.

bioRxiv preprint doi: https://doi.org/10.1101/2021.11.10.468116; this version posted November 13, 2025. The copyright holder for this preprint (which was not certified by peer review) is the author/funder, who has granted bioRxiv a license to display the preprint in perpetuity. It is made available under aCC-BY 4.0 International license.

Figure S2

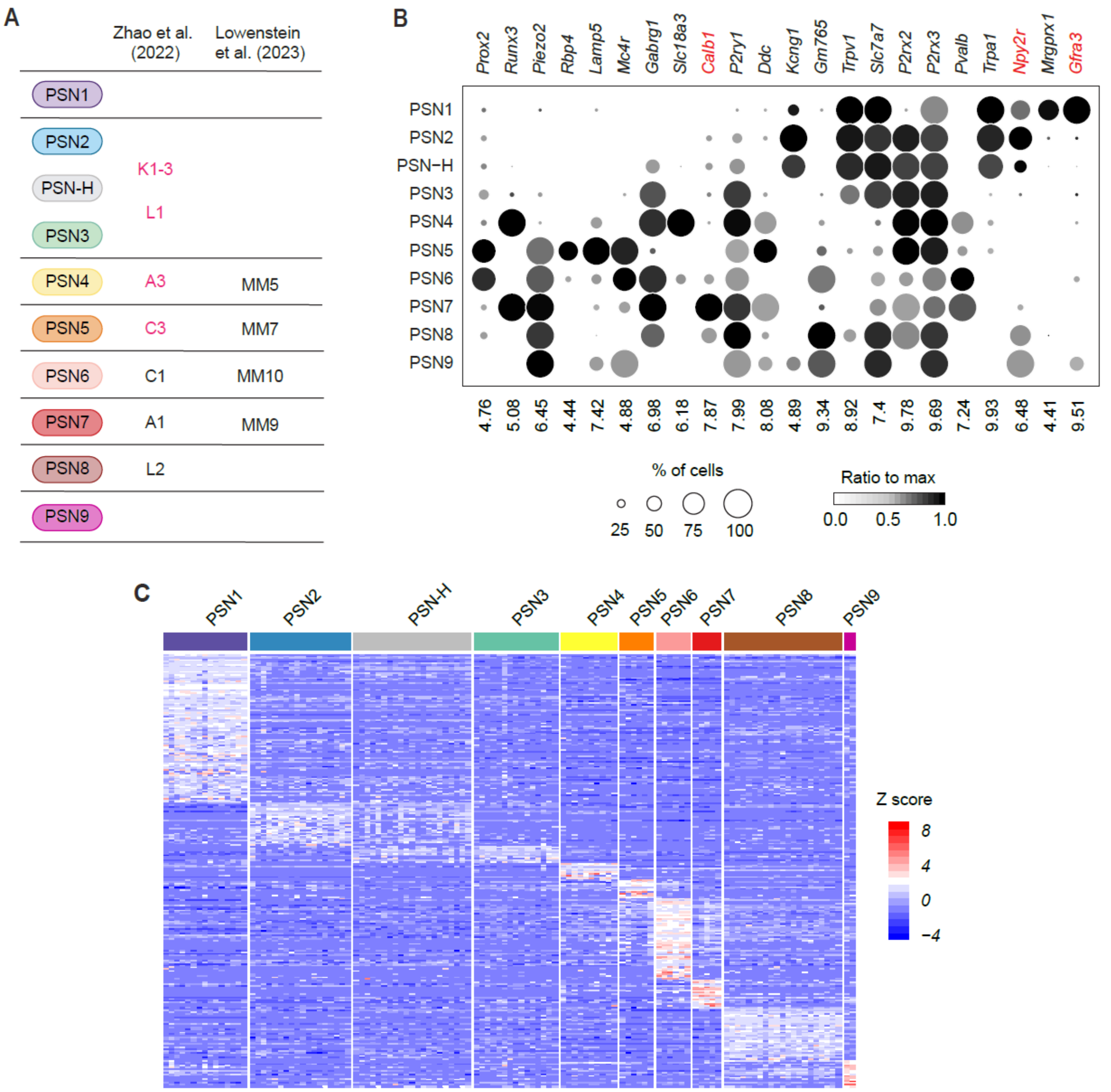

D

Subtype	Genes	No./total WGA+ cells	%	n (ganglia)	Method	Tissue type
PSN1	*Gal+*	48/620	7.7	3	ISH	Section
PSN2	*Npy2r+ Htr3b-*	143/772	18.5	3	ISH	Section
PSN-H	*Npy2r+ Htr3b+*	75/772	9.7*	3	ISH	Section
PSN3	*Npy2r- Htr3b+*	166/772	21.5**	3	ISH	Section
PSN4	*Slc18a3+*	67/832	8.1	5	ISH	Section
PSN5	*Lamp5+*	53/856	6.2	3	ISH	Section
PSN6	*Lmcd+*	45/556	8.1	3	ISH	Section
PSN7	Calb1+	164/2211	7.4	5	IHC	Whole-mount ganglion
PSN8	*Kcnv1+*	94/618	15.2	3	ISH	Section
PSN9	*Gal- Phox2b-*	15/620	2.5	3	ISH	Section

bioRxiv preprint doi: https://doi.org/10.1101/2021.11.10.468116; this version posted November 13, 2025. The copyright holder for this preprint (which was not certified by peer review) is the author/funder, who has granted bioRxiv a license to display the preprint in perpetuity. It is made available under aCC-BY 4.0 International license.

Figure S2. Molecular markers and abundances of PSN subtypes, related to Figure 2

(A) Matching PSN subtypes with vagal sensory clusters identified in two previously published scRNA-seq studies based on molecular marker expression shown in (B). Magenta clusters from Zhao et al. (2022) were identified as lung-innervating clusters.

(B) Expression levels of genes used for matching PSN subtypes in this study with previously described vagal sensory clusters or subpopulations of vagal PSNs. Red highlights genes previously identified as markers of subpopulations of vagal PSNs and enriched in single PSN subtypes in this study.

(C) Heatmap showing relative expression levels of 260 subtype-enriched genes across the 10 molecular subtypes. Z scores were calculated for individual genes across all cells.

(D) Quantification of ISH and immunostaining results using probes/antibodies for the subtype selective genes. Calb1 was detected by immunostaining on whole-mount ganglia, therefore yielded a significantly higher number of total WGA^{+} cells. *, likely lower than the true fraction of PSN-H neurons since *Npy2r* is expressed in a subset of these neurons. **, likely higher than the true fraction of PSN3 neurons for the same reason.

bioRxiv preprint doi: https://doi.org/10.1101/2021.11.10.468116; this version posted November 13, 2025. The copyright holder for this preprint (which was not certified by peer review) is the author/funder, who has granted bioRxiv a license to display the preprint in perpetuity. It is made available under aCC-BY 4.0 International license.

Figure S3

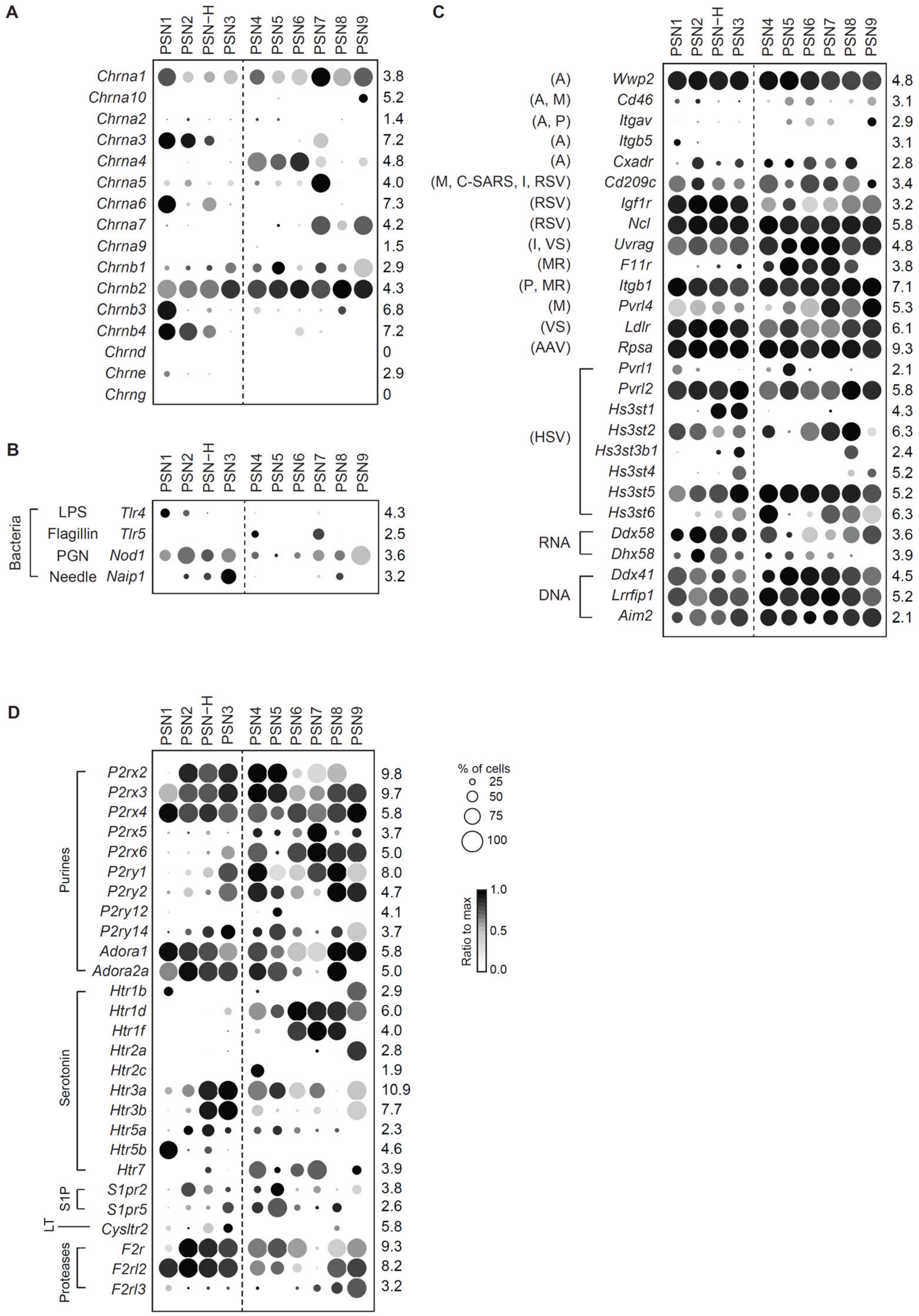

bioRxiv preprint doi: https://doi.org/10.1101/2021.11.10.468116; this version posted November 13, 2025. The copyright holder for this preprint (which was not certified by peer review) is the author/funder, who has granted bioRxiv a license to display the preprint in perpetuity. It is made available under aCC-BY 4.0 International license.

Figure S3. Functional receptors expressed in PSN subtypes, related to Figure 3

(A) Expression patterns of nicotinic acetylcholine receptor subunits across PSN subtypes.

(B) Expression of pathogen pattern recognition receptors for bacterial cellular components. LPS, lipopolysaccharide; PGN, peptidoglycan.

(C) Expression of entry receptors for respiratory viruses and intracellular nucleic acid sensors (magenta) across PSN subtypes. A, adenovirus; M, measles; P, parechovirus; C-SARS, SARS-coronavirus; I, influenza; RSV, respiratory syncytial virus; VS, vesicular stomatitis virus; MR, mammalian orthoreovirus; AAV, adeno-associated virus; HSV, herpes simplex virus.

(D) Expression of additional receptors for inflammatory mediators. S1P, sphingosine-1-phosphate; PG, prostaglandins; LT, leukotrienes.

bioRxiv preprint doi: https://doi.org/10.1101/2021.11.10.468116; this version posted November 13, 2025. The copyright holder for this preprint (which was not certified by peer review) is the author/funder, who has granted bioRxiv a license to display the preprint in perpetuity. It is made available under aCC-BY 4.0 International license.

Figure S4

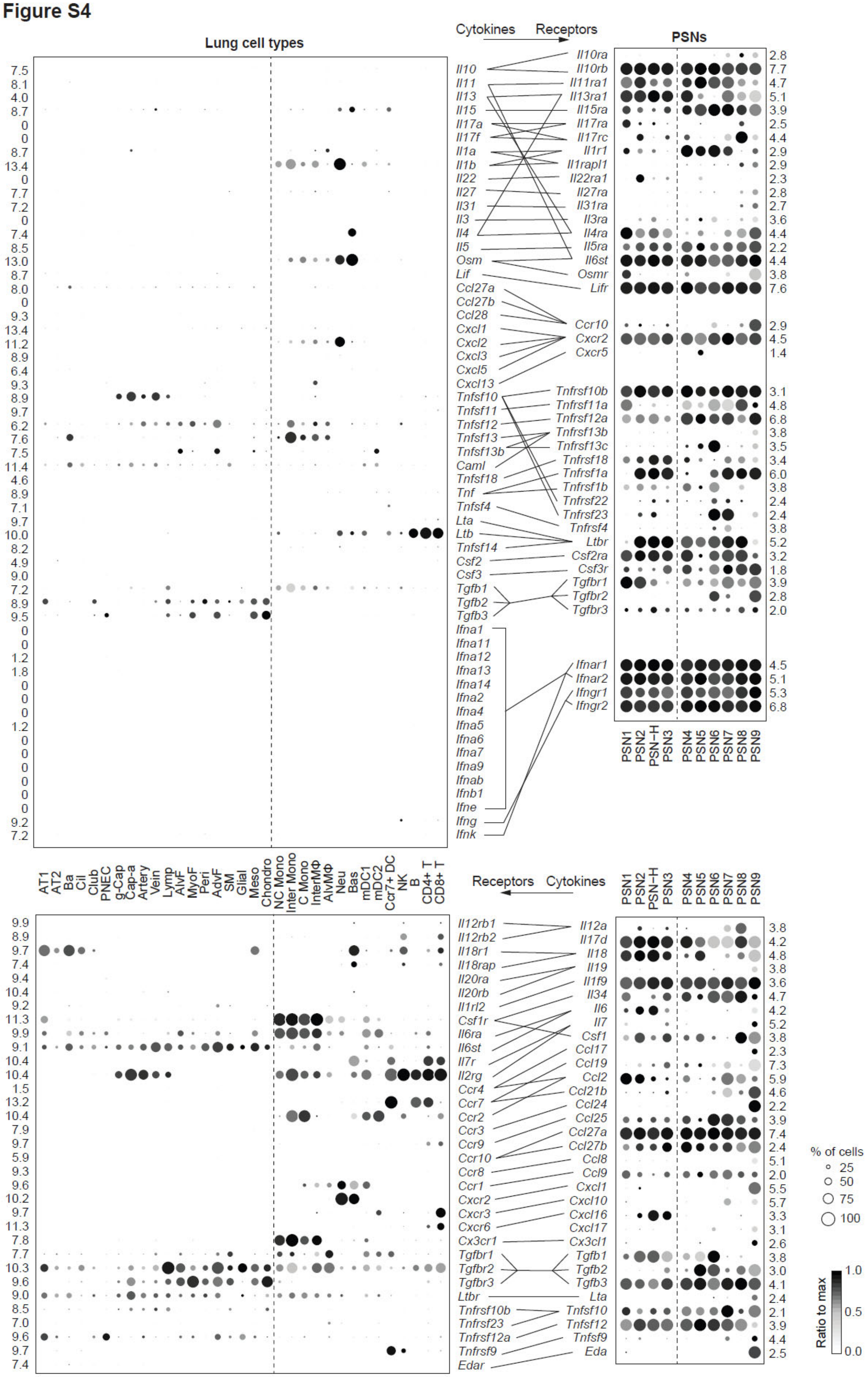

bioRxiv preprint doi: https://doi.org/10.1101/2021.11.10.468116; this version posted November 13, 2025. The copyright holder for this preprint (which was not certified by peer review) is the author/funder, who has granted bioRxiv a license to display the preprint in perpetuity. It is made available under aCC-BY 4.0 International license.

Figure S4. Local interactions between lung cells and PSNs, related to Figure 3

Predicted bi-directional cytokine signaling interactions between lung cells and vagal PSNs, inferred from expression of cytokine genes and the cognate receptor genes (connected by lines, arrows at top show the signaling direction). Full list of ligands and receptors screened is in Table S5, and genes expressed in >50% of neurons and a mean log2(TPM+1) > 1 in at least one PSN subtype are shown here. Dashed lines divide lung immune (right side) from non-immune (left) cell types (left dot plots), and Group I (left) from Group II (right) PSN subtypes (right dot plots). AT1, alveolar type 1 cells; AT2, alveolar type 2 cells; Ba, basal cells; Cil, ciliated cells; Club, club cells; PNEC, pulmonary neuroendocrine cells; Cap, general capillary endothelial cells (g-cap); Cap-a, capillary aerocytes; Artery, arterial endothelial cells; Vein, venous endothelial cells; Lymp, lymphatic endothelial cells; AlvF, alveolar fibroblasts; MyoF, myofibroblasts; Peri, pericytes; AdvF, adventitial fibroblasts; SM, smooth muscle cells; Glial, peripheral glial cells; Meso, mesothelial cells; Chondro, Chondrocytes; mDC1, myeloid dendritic type 1 cells; mDC2, myeloid dendritic type 2 cells; Ccr7+ DC, Ccr7+ dendritic cells; NC Mono, non-classical monocytes; inter Mono, intermediate monocytes; C Mono, classical monocytes; B, B cells; CD4+ T, CD4+ T cells; CD8+ T, CD8+ T cells; NK, natural killer cells; Neu, neutrophils; Bas, Basophils; InterMϕ, interstitial macrophages; AlvMϕ, alveolar macrophages.

bioRxiv preprint doi: https://doi.org/10.1101/2021.11.10.468116; this version posted November 13, 2025. The copyright holder for this preprint (which was not certified by peer review) is the author/funder, who has granted bioRxiv a license to display the preprint in perpetuity. It is made available under aCC-BY 4.0 International license.

Figure S5

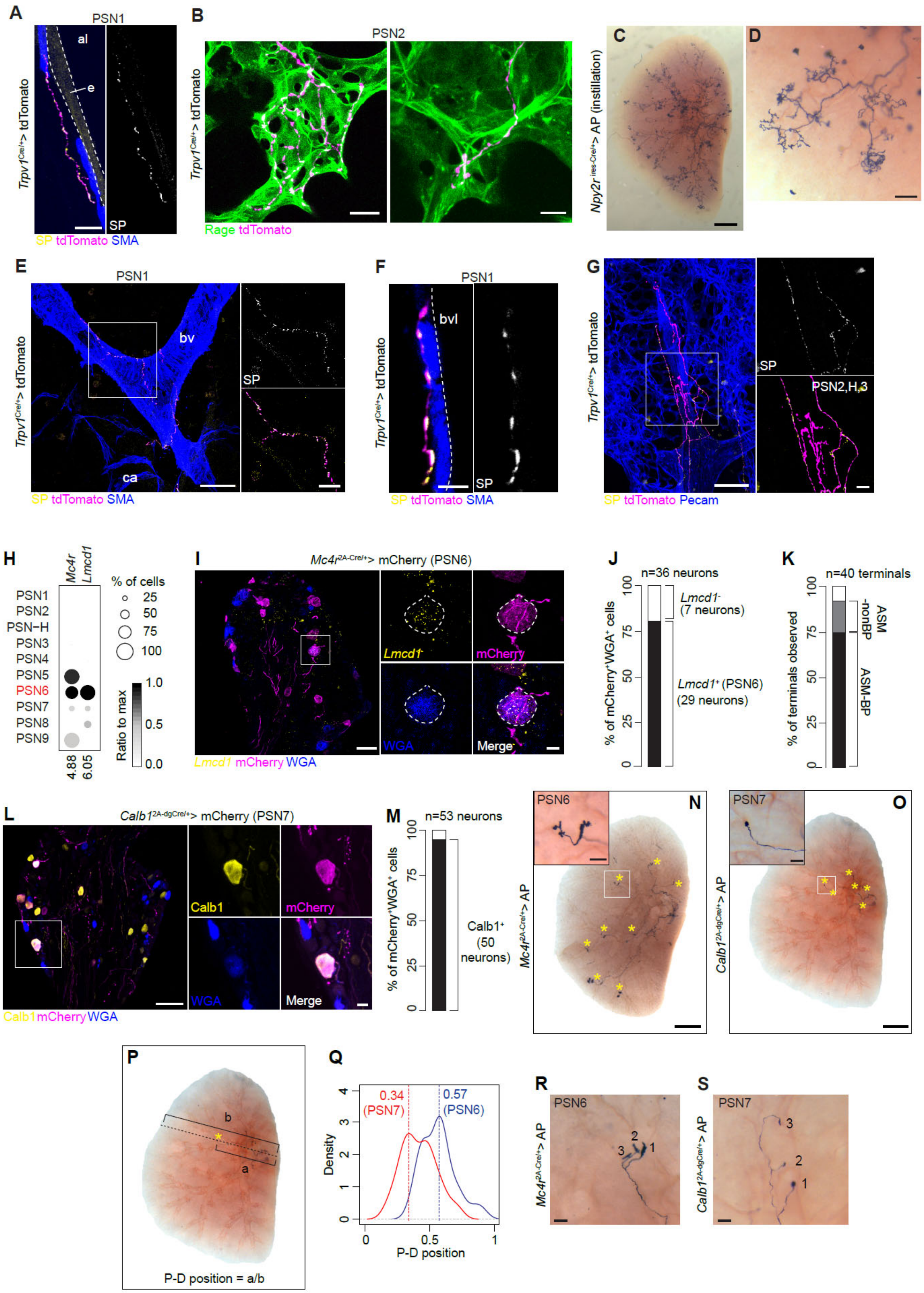

bioRxiv preprint doi: https://doi.org/10.1101/2021.11.10.468116; this version posted November 13, 2025. The copyright holder for this preprint (which was not certified by peer review) is the author/funder, who has granted bioRxiv a license to display the preprint in perpetuity. It is made available under aCC-BY 4.0 International license.

Figure S5. Terminal locations and morphologies of PSN subtypes, related to Figure 4

(A) Immunostaining of a lung section from *Trpv1*$^{Cre/+}$ mice injected with AAV-CAG-DIO-tdTomato into the vagal ganglia, showing a SP+tdTomato+ fiber (PSN1) runs beneath the airway smooth muscle stained for smooth muscle actin (SMA). Note gaps between smooth muscle fibers on conducting airways, which likely allow sensing of environmental chemicals just penetrating epithelium or molecules secreted by epithelial cells. e, epithelium (outlined); al, airway lumen. Scale bar: 20 µm.

(B) Examples of alveolar terminals (likely PSN2) elaborating on the surface of an air sac (left) and between air sacs (right, more common). Scale bars: 10 µm.

(C, D) Whole-mount alkaline phosphatase (AP) staining of lung lobes from *Npy2*$^{rires\text{-}Cre/+}$ mice intratracheally instilled with the Cre-dependent AP virus (labels PSN2). Note in D, all branches of the labeled fibers terminate in the alveolar region but not on the conducting airways. Scale bars: 1 mm (C), 0.1 mm (D).

(E, F) SP+tdTomato+ fibers (PSN1) terminate on pulmonary blood vessels (bv). ca, conducting airway. F shows a fiber running beneath the vascular smooth muscle. Scale bars: 100 µm (E), 20 µm (E inset), 5 µm (F).

(G) SP-tdTomato+ fibers (PSN2, H, or 3) also terminate on pulmonary blood vessels (endothelial cells stained by Pecam, blue). Scale bars: 50µm, 10 µm (inset).

(H) Expression patterns of *Mc4r* and PSN6 marker *Lmcd1* across PSN subtypes.

(I) ISH of PSN6 subtype marker *Lmcd1* with mCherry and WGA double immunostaining on a vagal ganglion section from a *Mc4r*$^{2A\text{-}Cre/+}$ mouse injected with AAV-Syn-DIO-hM3Dq-mCherry into the ganglion. Close-up of the boxed area is shown in right panels with channels split; outlined neuron is an example of labeled PSN6 neuron. Scale bars: 50µm, 10µm (inset).

bioRxiv preprint doi: https://doi.org/10.1101/2021.11.10.468116; this version posted November 13, 2025. The copyright holder for this preprint (which was not certified by peer review) is the author/funder, who has granted bioRxiv a license to display the preprint in perpetuity. It is made available under aCC-BY 4.0 International license.

(J) Quantification of PSN6 labeling specificity (serial sections of two ganglia from two *Mc4r*$^{2A\text{-}Cre/+}$ mice). Lmcd1- labeled neurons are likely PSN5 neurons.

(K) Locations of labeled termini in *Mc4r*$^{2A\text{-}Cre/+}$ mice injected with reporter virus into the ganglia. Quantifications were done by examining all vibratome sections of 4 entire left lobes from 4 mice. ASM, airway smooth muscle; BP, branch point.

(L) Immunostaining of PSN7 marker Calb1 with mCherry and WGA on a vagal ganglion section from a *Calb1*$^{2A\text{-}dgCre/+}$ mouse injected with AAV-Syn-DIO-hM3Dq-mCherry into the ganglion. Close-up of the boxed area with a labeled PSN7 neuron is shown in right panels with channels split. Scale bars: 50 μm, 10 μm (inset).

(M) Quantification of PSN7 labeling specificity (serial sections of 3 ganglia from 2 *Calb1*$^{2A\text{-}dgCre/+}$ mice).

(N, O) Whole-mount AP staining of right cranial lung lobes from *Mc4r*$^{2A\text{-}Cre/+}$ (left) and *Calb1*$^{2A\text{-}dgCre/+}$ (right) mice injected with AAV-CMV-FLEX-PLAP into the vagal ganglia. Asterisks mark termini in each lobe. Insets are close-ups of boxed regions showing terminal morphology. Scale bars: 1 mm, 0.1 mm (insets).

(P) Method of calculating proximal-distal (P-D) position of PSN termini. A line between the primary bronchus entry point into the lobe and a given terminal was drawn and extended to the edge of the lobe. Terminal P-D position is the ratio of the segment length between the entry point and the terminal to the total length of the line.

(Q) Kernel density plot showing distributions of PSN6 (blue) and PSN7 (red) terminal P-D positions (n=29 PSN6 termini, n= 36 PSN7 termini). Note PSN7 termini are distributed more proximally than PSN6 termini.

(R, S) Close-ups from whole-mount AP staining showing termini of single PSN6 (S) and PSN7 (T) fibers.

bioRxiv preprint doi: https://doi.org/10.1101/2021.11.10.468116; this version posted November 13, 2025. The copyright holder for this preprint (which was not certified by peer review) is the author/funder, who has granted bioRxiv a license to display the preprint in perpetuity. It is made available under aCC-BY 4.0 International license.

Individual termini are numbered. Scale bar: 0.1 mm. We cannot exclude the possibility that these axons also terminate in other lung lobes or organs.

bioRxiv preprint doi: https://doi.org/10.1101/2021.11.10.468116; this version posted November 13, 2025. The copyright holder for this preprint (which was not certified by peer review) is the author/funder, who has granted bioRxiv a license to display the preprint in perpetuity. It is made available under aCC-BY 4.0 International license.

Figure S6

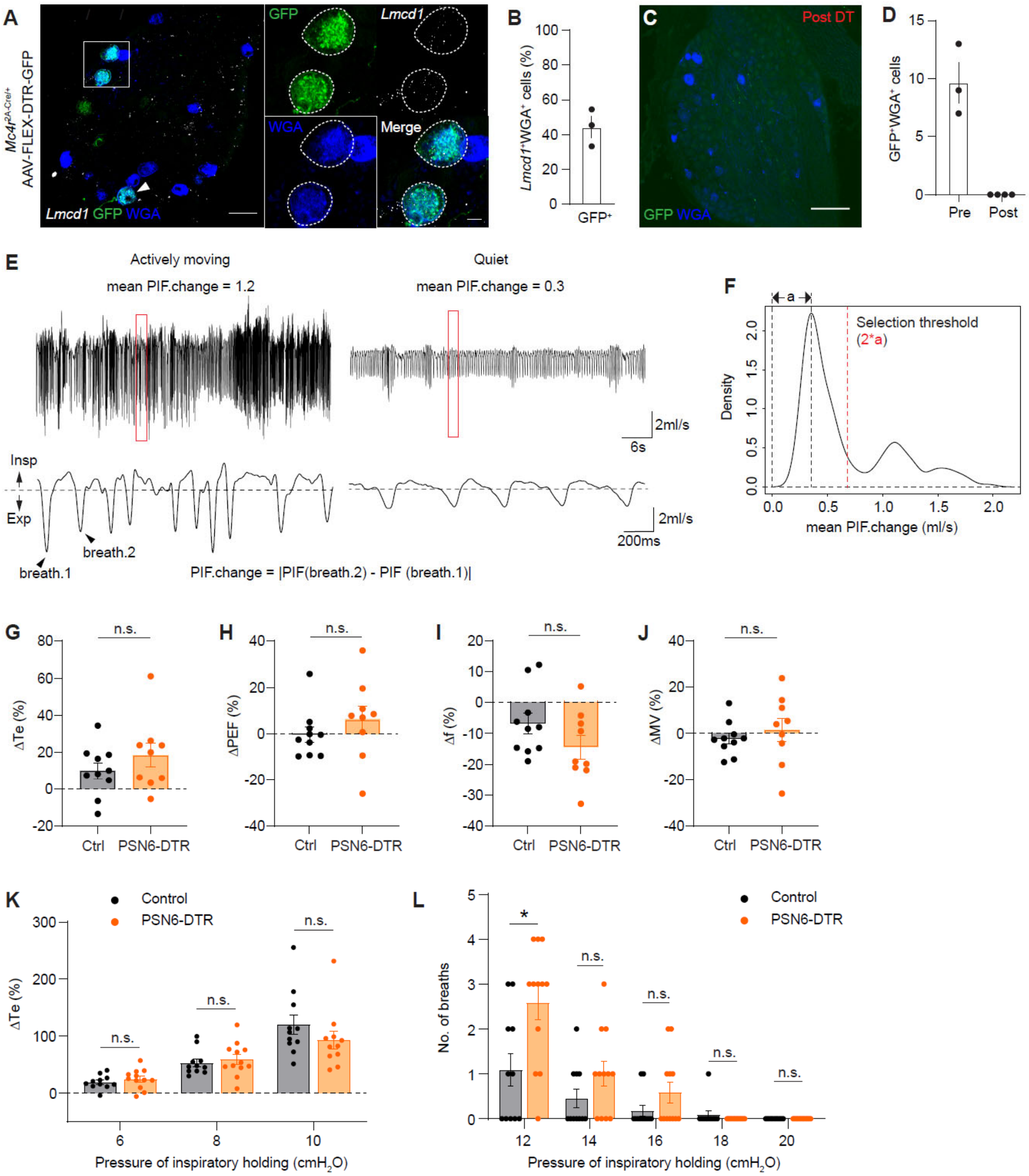

bioRxiv preprint doi: https://doi.org/10.1101/2021.11.10.468116; this version posted November 13, 2025. The copyright holder for this preprint (which was not certified by peer review) is the author/funder, who has granted bioRxiv a license to display the preprint in perpetuity. It is made available under aCC-BY 4.0 International license.

Figure S6. PSN6 ablation and breathing changes, related to Figure 6

(A) ISH of PSN6 subtype marker *Lmcd1* with GFP and WGA double immunostaining on a thin vagal ganglion section from a *Mc4r*$^{2A\text{-}Cre/+}$ mouse injected with AAV-CBA-FLEX-DTR-GFP into the ganglia. WGA was instilled into the lung to label PSNs. Panels at right are split channels of the boxed region showing two DTR-GFP expressing PSN6 neurons. Arrowhead, another DTR-GFP expressing PSN6 neuron. Scale bars: 50 μm and 10 μm (inset).

(B) Quantification of the percentage of *Lmcd1*+WGA+ PSN6 neurons expressing DTR-GFP in PSN6-DTR mice (n=57 *Lmcd1*+WGA+neurons scored on 3 ganglia from 3 mice, with each dot representing the result from all serial sections of one ganglion).

(C) Immunostaining for GFP and WGA in a ganglion section from a PSN6-DTR mouse three days after DT injection.

(D) Quantification of DTR-GFP expressing neurons in vagal ganglia of PSN6-DTR mice before and after DT injection (each dot represents the result from all serial sections of one ganglion).

(E) Selection of quiet breathing periods. Representative airflow traces during actively moving (left) or quiet (right) phases, recorded by whole body plethysmography. Portions of traces highlighted by red boxes are enlarged below. PIF.change is the absolute difference of peak inspiratory flows of two adjacent breaths. Mean PIF.change is the average PIF.change in one minute period. Note the large difference in this parameter between active (1.2 ml/s) and quiet (0.3 ml/s) breathing phases. Dashed lines, zero flow; Insp, inspiration; Exp, expiration.

(F) Kernel density plot showing distribution of mean PIF.change for every minute over the entire recording period. Time periods with mean PIF.change less than twice the PIF.change at the first peak (a) were selected as quiet breathing periods.

bioRxiv preprint doi: https://doi.org/10.1101/2021.11.10.468116; this version posted November 13, 2025. The copyright holder for this preprint (which was not certified by peer review) is the author/funder, who has granted bioRxiv a license to display the preprint in perpetuity. It is made available under aCC-BY 4.0 International license.

(G-J) Changes in expiratory time (Te, panel G), peak expiratory flow (PEF, panel H), respiratory frequency (f, panel I), and minute ventilation (MV, panel J) distribution modes after DT injection (post), normalized to before DT injection (pre) values, in control and PSN6-DTR mice.

(K) Changes in Te in control and PSN6-DTR mice after DT injections. ΔTe represents the change in the average time between EMG bursts during the first 2 seconds of inspiratory holdings at a given pressure, normalized to the average time between EMG bursts during the first 2 seconds of expiratory holding. Two-way ANOVA with sidak multiple comparison correction.

(L) Number of breaths during the first 2s of inspiratory holdings at different pressures in control and PSN6-DTR mice after DT injections. *p=0.0496, two-way ANOVA with sidak multiple comparison correction.

bioRxiv preprint doi: https://doi.org/10.1101/2021.11.10.468116; this version posted November 13, 2025. The copyright holder for this preprint (which was not certified by peer review) is the author/funder, who has granted bioRxiv a license to display the preprint in perpetuity. It is made available under aCC-BY 4.0 International license.

Figure S7

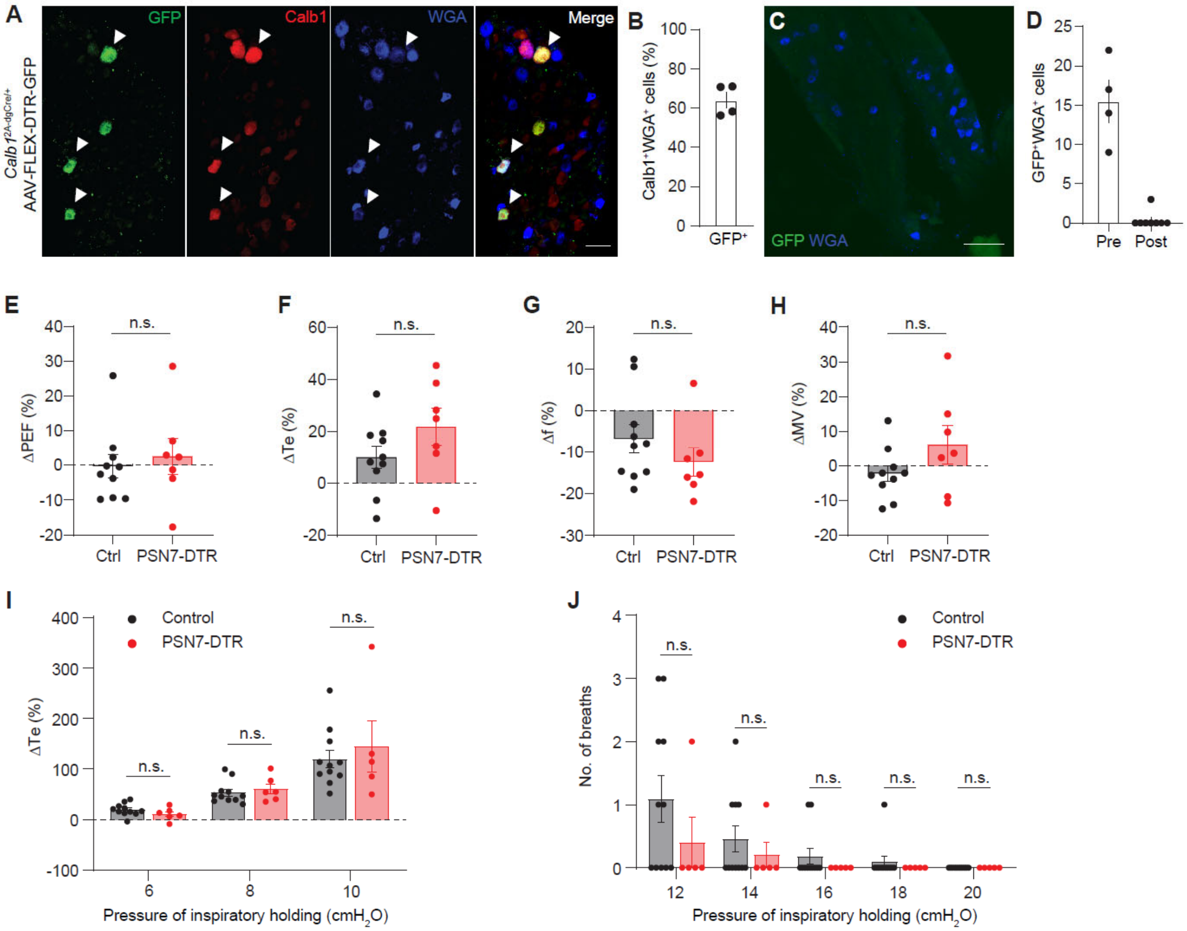

bioRxiv preprint doi: https://doi.org/10.1101/2021.11.10.468116; this version posted November 13, 2025. The copyright holder for this preprint (which was not certified by peer review) is the author/funder, who has granted bioRxiv a license to display the preprint in perpetuity. It is made available under aCC-BY 4.0 International license.

Figure S7. PSN7 ablation and breathing changes, related to Figure 7

(A) Immunostaining of PSN7 subtype marker Calb1 along with GFP and WGA on a thin vagal ganglion section from a *Calb1*$^{2A\text{-}dgCre/+}$ mouse injected with AAV-CGA-FLEX-DTR-GFP into the ganglion and treated with TMP. WGA was instilled into the lung to label PSNs. Arrowheads, DTR-GFP expressing PSN7 neurons. Scale bar: 50 µm.

(B) Quantification of the percentage of Calb1^{+}WGA^{+} PSN7 neurons expressing DTR-GFP in PSN7-DTR mice (n= 95 Calb1^{+}WGA^{+} neurons scored from 4 ganglia of 2 mice, with each dot representing result from all serial sections of one ganglion).

(C) Immunostaining for GFP and WGA on a ganglion section from a PSN7-DTR mouse three days after DT injection.

(D) Quantification of DTR-GFP expressing PSNs in vagal ganglia of PSN7-DTR mice before (pre) and after (post) DT injection (each dot represents the result from all serial sections of one ganglion).

(E-H) Changes in expiratory time (Te, panel G), peak expiratory flow (PEF, panel H), respiratory frequency (f, panel I), and minute ventilation (MV, panel J) distribution modes after DT injection (post), normalized to before DT injection (pre) values, in control and PSN7-DTR mice.

(I) Changes in Te in control and PSN7-DTR mice after DT injections. ΔTe represents the change in the average time between EMG bursts during the first 2 seconds of inspiratory holdings at a given pressure, normalized to the average time between EMG bursts during the first 2 seconds of expiratory holding. Two-way ANOVA with sidak multiple comparison correction.

(J) Number of breaths during the first 2s of inspiratory holdings at different pressures in control and PSN7-DTR mice after DT injections. Two-way ANOVA with sidak multiple comparison correction.
